# The Gut–Brain-Axis as a Target to Treat Stress-Induced Obesity

**DOI:** 10.3389/fendo.2014.00117

**Published:** 2014-07-18

**Authors:** Chooi Yeng Lee, Alfonso Abizaid

**Affiliations:** ^1^School of Pharmacy, Monash University Malaysia, Bandar Sunway, Malaysia; ^2^Department of Neuroscience, Carleton University, Ottawa, ON, Canada

**Keywords:** gut, obesity, brain, stress, gut peptides

The emergence of obesity as a pandemic has led to increased efforts to determine the causes for this disorder and potential new treatments to prevent and/or treat those affected by it. The mechanisms underlying the ontogeny of obesity are complex. They involve an interaction between a genetic predisposition to this disorder and environmental conditions that catalyze the development of an obese phenotype (1). It has become evident that stress may be a strong environmental factor leading to metabolic changes that lead to obesity. Stress is a concept coined to describe the state that is generated when physiological or psychological wellbeing is challenged. It is associated with physiological and behavioral responses considered adaptive and conducive to reduce or to cope with the challenges posed by the stressor (2, 3). Continuous stressful events, however, result in pathological states including some of the same conditions associated with obesity (4, 5). In particular, continuous social stressors result in increased body weight and abdominal fat deposition, insulin resistance, and cardiovascular disease (2, 4). The underlying mechanisms and relevant potential treatments are less well documented. Nevertheless, there is increasing evidence to suggest that psychological stressors represent a homeostatic challenge, and as such have a strong impact on the brain systems associated with homeostatic control (3).

It is clear that the brain plays a critical role in the regulation of energy balance, and as such represents a target for therapeutic intervention. For instance, cells groups within the hypothalamic arcuate nucleus (ARC) are important for regulating food intake and energy balance, whereas a number of regions across the mesolimbic dopaminergic system and ascending noradrenergic inputs stemming from the brain stem regulate hedonic and short term feeding responses (6). In spite of this, few viable therapeutic options have emerged from these advances particularly given the fact that many drugs targeting these systems have substantial side effects. Here, we propose that the periphery, and in particular the gut, may represent an alternate target for treatments that can reduce obesity, particularly in the face of stress.

## Regulation of Food Intake and Energy Balance by Gut Hormones

Recent evidence has brought greater attention to the gut as a key contributor to the regulation of food intake and energy balance. The gut serves both as a sensory organ for nutrients and can regulate the activity of brain centers associated with the regulation of food intake and energy balance. One indication of the importance of the gut–brain-axis is that gastrointestinal cells serve as nutrient sensors and produce hormonal and neural responses to nutrients that target the brain to modulate food intake and energy balance (7). A number of experiments have demonstrated that animals can detect and bar press for intragastric infusions of solutions containing sucrose or fat infused directly into the gut (7). The presence of lipids in the gut decreases hepatic glucose production, linking the gut–brain-axis with liver function (8). Vagotomy, inhibition of the *N*-methyl-d-aspartate receptor in the nucleus of the solitary tract, sympathetic denervation, and blockade of β_2_-adrenoceptor abolished the effects of lipid on the regulation of glucose homeostasis (9). Notably, the gut–brain–liver-axis is disturbed by chronic exposure to a high fat diet (10). In addition to stimulating the ascending vagus nerve, cells in the gut signal the brain through a number of endocrine signals. These include peptide YY (PYY), neuropeptide Y (NPY), cholecystokinin (CCK), oxyntomodulin (OXM), glucagon-like peptide-1 (GLP1), and ghrelin, all of which control appetite and glucose homeostasis (11–14). While PYY, CCK, OXM, and GLP1 are anorectic and some increase energy expenditure, ghrelin is a potent orexigenic hormone that also influences metabolic rate by favoring the utilization of carbohydrates instead of lipids as a source of energy, resulting in increased adiposity and body weight (15). Given that stressors and the physiological responses elicited to cope with them can generate a substantial energy drain, it is not surprising that these gut signals are altered during the stress response, and hence could represent a novel target to control stress-induced obesity.

## Stress and the Gut–Brain-Axis

The effects of stress on the gastrointestinal system have been known for a long time, in particular the effects of stress on gastric motility and on gastric acid secretion. Continuous stress has been associated with gastric ulceration and other gastrointestinal disorders like irritable bowel syndrome. One would presume that, if the gut plays an important role in the regulation of energy balance, and if the function of the gut is altered during stress, then stress could alter the function of the gut to promote obesity. The clearest evidence for this is the effect of stress on ghrelin secretion. Ghrelin, a 28 amino acid peptide secreted by oxyntic cells in the stomach and upper intestine, is the only gastrointestinal peptide known to stimulate food intake and alter energy expenditure (15, 16). Plasma ghrelin rises following an acute fast or during periods of caloric restriction, where the daily intake of accessible food is lower than the daily *ad libitum* access (17). Interestingly, ghrelin is secreted concomitantly with glucocorticoids following acute and chronic stress (18, 19) and plasma active ghrelin concentrations remain significantly elevated in the late phase of a stress session (18).

Acute stressors elevate plasma ghrelin through the activation of the sympathetic and enteric nervous system, but recent data suggest that stress-induced ghrelin secretion may be the result of stimulation of corticotropin releasing hormone (CRH) receptors in the gut, CRH and CRH-related peptides such as urocortin-1 and 2 (20–22). For instance, central stimulation of CRH1 and CRH2 receptors produces stress like effect in gastrointestinal motility, gastric emptying, and colonic propulsion, whereas blockade of CRH1 and CRH2 receptors prevents some of these effects (22). Ghrelin secretion in response to stress may also be related to the effects of locally released urocortin-1 acting on CRH2 receptors in the gut (21). This process may be important acutely, given that urocortin reduces pain in the gastrointestinal tract (23), and given that ghrelin protects the stomach against gastric ulceration induced by repeated stressors (24–26). Problems, however, may arise when the stressor is chronic. For instance, in mice, chronic social defeat stress regimen that lasts 10–21 days, increases ghrelin concentrations in concert with increases in caloric intake and weight gain (27, 28). This stress paradigm also increases hypothalamic expression of orexigenic peptides such as NPY and Agouti-related peptide, and plasma biomarkers indicative of obesity, an effect that persisted for at least 2 weeks after the stress paradigm was terminated (27). In contrast, GHSR KO mice or mice receiving chronic intracerebroventricular infusions of a ghrelin receptor antagonist do not increase their caloric intake or weight gain in response to the same stressor (27). Thus, it is clear that prolonged periods of social stress can lead to high ghrelin concentrations that promote higher caloric intake and alterations in energy expenditure that lead to weight gain and adipose tissue accumulation.

Another mechanism by which stress-induced ghrelin secretion is the stimulation of the mesolimbic dopaminergic system that is critical for the regulation of reward seeking behaviors. Ghrelin receptors are found in dopamine neurons within the midbrain ventral tegmental area (VTA), and here ghrelin can stimulate dopamine release and food intake and motivation to obtain palatable foods, and ghrelin receptor antagonism prevents this (1, 29, 30). Similarly, mice with genetic deletion of the GHSR show less preference for high calorie foods. Selectively restoring ghrelin receptors in dopamine producing cells can enhance their preference for these foods (31, 32). During stress, ghrelin may act in the VTA to increase appetite, but prolonged exposure to stressors may ultimately prevent ghrelin from increasing appetite in this region and ultimately lead to anhedonia (28, 31). Given these data, ghrelin, urocortin-1, and their respective receptors represent promising potential peripheral targets to reduce stress-induced weight gain and appetite.

Besides ghrelin, other gut peptides are also secreted and may have an influence in the stress response, although less is known about how prolonged periods of stress affect the secretion of these peptides. Acute stressors like restraint cause increases in the peripheral and central release of NPY, GLP1, CCK, OXM, and motilin (33–35). Of these, NPY has received special attention for a number of reasons. NPY neurons in the ARC are important in the integration of peripheral signals regulating energy balance including those coming from the gut, and project to hypothalamic and extrahypothalamic brain region to stimulate feeding and to alter behavior including those associated with mood (36, 37). Sympathetic nervous system terminals also release NPY. Following chronic stress, increased sympathetic release of NPY leads to inflammatory responses, fat angiogenesis, and adipocyte enlargement and proliferation ultimately leading to obesity, and these effects are mediated by Y_2_ receptors localized in adipocytes (38). It is not known if gut derived NPY is over-secreted following chronic stress, or if it has similar direct effects on adipocytes as NPY secreted by sympathetic terminals, but it is not unlikely that this would contribute to an obesogenic state.

A hormone that could counter the NPY effects is GLP1. This peptide is released by L-cells in the gut and has emerged as an important player in the regulation of appetite and glucose homeostasis (39). In addition, GLP1 is released centrally and acts both in the hypothalamus and midbrain VTA dopamine cells to reduce appetite, increase energy expenditure, and decrease motivated behaviors (40–43). Interestingly, GLP1 KO mice have abnormal hormonal responses to acute stressors (44). Within the periphery, GLP1 can act locally to protect the gut from stress-induced gastric acid secretion, and is important for altering gastric motility (45, 46). More importantly, GLP1 protects a number of tissues affected by chronic stress including pancreatic β-cells, cardiomyocytes, and kidney cells, while reducing cytokine induced inflammation (47–50). Whether chronic stress results in altered secretion of either of these peptides is not known, and it may be critical to determine if this is the case in order to fully determine the usefulness of these peptides as potential treatments for stress-induced pathology.

## Potential Future Directions

One of the problems that exist with trying to counter pathological conditions associated with stress is that either the stressor is difficult to remove or the stressor leaves symptoms that persist in spite of the stressor being removed. In this sense, pharmacological interventions derived from gut peptides and aimed at reducing metabolic alterations caused by stress may not represent a “magic bullet” that can reverse metabolic changes to an optimal state. These, however, may become interventions that can help to deal with these problems in the short term while a patient finds a way to deal or remove herself from the stressor. For example, drugs that act to decrease ghrelin signaling (i.e., ghrelin receptor antagonists or inverse agonists) could be used to decrease stress-induced caloric intake and weight gain as well as stress-induced gastric alterations (51, 52). Nevertheless, enthusiasm for these types of drugs has been hampered by evidence suggesting that stress-induced ghrelin secretion is necessary not only to maintain metabolic homeostasis but also to prevent stress-induced depressive like behaviors and reduce anxiety (28, 53, 54). These data remain unclear, however, as other studies show that ghrelin is actually anxiogenic and increases the formation of fearful memories (55–59). Clearly, an in depth analysis of these data is required to explain these paradoxical results, but at the very least, they suggest that drugs blocking the ghrelin system could have a negative impact on mood. Furthermore, the use of ghrelin receptor antagonists or inverse agonists may cause undesired side effects given the ligand independent interaction between ghrelin receptors and other G-coupled protein receptors in the central nervous system (60).

Perhaps a better alternative would be to use drugs that decrease acyl-ghrelin levels without depleting the system from ghrelin, or altering GHSR signaling thereby maintain ghrelin’s protective effects. One potential target for this is ghrelin-*O*-acyltransferase (GOAT, also known as MBOAT4), an enzyme that is required for the esterification process that links *n*-octanoic acid to the ghrelin molecule (61). The GOAT enzyme is produced by the same cells that secrete ghrelin (61), and drugs that reduce the activity of this enzyme not only reduce plasma active (acylated) ghrelin concentrations, but they also cause a decrease in weight gain and adiposity in mice (62). Whether GOAT inhibitors improve metabolic changes caused by stressors remains to be determined. Alternatively, des-acyl ghrelin may also be useful given that, like GOAT inhibitors, des-acyl ghrelin and its analogs decrease acyl-ghrelin concentrations, decrease high fat diet intake, weight gain, and adiposity, improve glycemic index, and are protective in cardiomyocytes in a GHSR independent manner (63–65). Finally, CRH2 receptor antagonists could be used to prevent stress-induced release of ghrelin to prevent the over-secretion of this peptide.

Nevertheless, GLP1 may be the most viable target at the moment since a number of analogs for this peptide are already FDA approved and currently used in the control of type II diabetes. Thus, drugs that mimic GLP1 or that decrease the activity of dipeptidyl-peptidase IV, an enzyme that cleaves GLP1 into an inactive byproduct, may be useful in increasing incretin tone and reducing the effects of stress on metabolism by doing so. GLP1 treatments may, however, be most useful when acting peripherally and not centrally, as GLP1 and its analogs can exacerbate stress responses and decrease motivated behaviors acting in the brain (66, 67). This, however, may not be the case as a GLP1 analog that crosses the blood brain barrier did not have an anxiogenic effect, and increased hippocampal neurogenesis (68).

In conclusion, it is only through identifying and understanding the mechanisms responsible for stress-induced obesity that effective therapeutics can be generated. Gut peptides associated with hunger and satiety may represent important players in these mechanisms, as they are also modulated by the responses to stressors. More importantly, they may also represent a potential therapeutic avenue for acute pharmacological intervention given that these are produced peripherally, and also influence the central nervous system. Nevertheless, relatively speaking, little is known about how these peptides are regulated in the face of stress, particularly chronic stressors. This knowledge is critically needed to determine if these peptides and their receptors will be useful for the treatment of stress-induced pathological conditions including obesity and metabolic syndrome.

## Conflict of Interest Statement

The authors declare that the research was conducted in the absence of any commercial or financial relationships that could be construed as a potential conflict of interest.
